# Women Planned for Immediate Lymphatic Reconstruction During Axillary Lymph Node Dissection Should Be Reconstructable: Improving Intraoperative Team Collaboration

**DOI:** 10.1177/22925503251404050

**Published:** 2025-12-19

**Authors:** Spencer Yakaback, Rosalie Morrish, Golpira Elmi Assadzadeh, Antoine Bouchard-Fortier, Alexandra Hatchell, Jennifer Matthews, Claire Temple-Oberle

**Affiliations:** 1 Department of Surgery, 2129 University of Calgary, Calgary, AB, Canada; 2 Cumming School of Medicine, University of Calgary, Calgary, AB, Canada; 3 Department of Oncology, University of Calgary, Calgary, AB, Canada

**Keywords:** immediate lymphatic reconstruction, breast cancer-related lymphedema, lymphatic reconstruction, supermicrosurgery, reconstruction lymphatique immédiate, lymphœdème lié au cancer du sein, reconstruction lymphatique, supermicrochirurgie.

## Abstract

**Introduction:** Immediate lymphatic reconstruction (ILR) during axillary lymph node dissection (ALND) has been shown to reduce breast cancer-related lymphedema (BCRL). However, some authors report many “non-reconstructable” patients, meaning that there were no suitable lymphatics or veins available in the axilla to complete the reconstructive procedure once the extirpative portion was complete. In contrast, almost all of our patients planned for ALND/ILR have had appropriate donor and recipient vessels. The purpose of our study was to contrast the incidence of “non-reconstructable” patients in the literature with our experience and highlight tips to improve the reconstructable rate. **Methods:** Step 1: A systematic review identified publications on ILR during ALND, which reported the number of “non-reconstructable” patients. Step 2: A chart review of ILR cases at the University of Calgary was conducted. From both data sets, patient demographics, cancer stage, node dissection results, treatment details and operative details were collected. The data was then analyzed to identify factors that could contribute to the number of “non-reconstructable” patients. **Results:** 11 studies were identified in the review, which included 949 patients planned for ILR during ALND. One hundred and thirty-three (14%) were deemed “non-reconstructable,” and did not undergo ILR. Analysis of 68 consecutive ALND/ILR cases at the University of Calgary identified 4 (5.9%, *p = .*03) “non-reconstructable” patients. A similar method of lymphatic mapping was used in the review studies as at the University of Calgary. The patients’ demographics and treatment details were similar in the review and our prospective series: average age (49 vs 54, *p = .*07, BMI (27 vs. 27, *p = .*47) and receipt of radiation (76.5% vs. 69%, *p = .*79). The only difference noted was the presence and the level of involvement of a plastic surgeon throughout the extirpative portion of the procedure, in order to identify and preserve vessels. At our institution, the plastic surgeon attends throughout and participates in a well-coordinated “dance” between the oncologic surgeon and the plastic surgeon. This coordination was not described in any of the studies reviewed. **Conclusions:** For ILR, coordinated plastic surgical involvement during ALND may reduce the number of “non-reconstructable” patients.

## Résumé

**Introduction :** Il a été démontré que la reconstruction lymphatique immédiate (RLI) lors d’une dissection des ganglions axillaires (DGA) réduit le lymphœdème lié au cancer du sein. Cependant, certains auteurs rapportent un grand nombre de patientes « non reconstructibles », c’est-à-dire qu’aucun vaisseau lymphatique ou veine adéquat n’était disponible dans l’aisselle pour réaliser la reconstruction une fois la partie extirpative a été terminée. En revanche, presque toutes nos patientes prévues pour une DGA/RLI disposaient de vaisseaux donneurs et receveurs appropriés. Cette étude visait à comparer l’incidence de patientes « non reconstructibles » dans la littérature avec notre expérience et à proposer des stratégies pour augmenter le taux de reconstructibilité. **Méthodes :** Étape 1 : Une revue systématique a identifié des publications sur la RLI lors de DGA, rapportant le nombre de patientes « non reconstructibles ». Étape 2 : Une analyse rétrospective des dossiers des cas d'ILR à l'Université de Calgary a été réalisée. Les deux ensembles de données ont permis de recueillir les caractéristiques démographiques des patientes, le stade du cancer, les résultats de la dissection ganglionnaire, ainsi que les détails du traitement et de l’intervention. Les données ont ensuite été analysées pour déterminer les facteurs influençant le nombre de patientes « non reconstructibles ». **Résultats :** La revue exploratoire a identifié 11 études portant sur 949 patientes prévues pour une RLI lors d’une DGA. 133 patientes (14%) ont été considérées « non reconstructibles » et n'ont pas bénéficié d'une RLI. L'analyse de 68 cas consécutifs de DGA/RLI à l'Université de Calgary a identifié 4 patientes (5.9%, *p = *0,03) « non reconstructibles ». Une méthode similaire de cartographie lymphatique a été utilisée dans les études de la revue, comme à l’Université de Calgary. Les caractéristiques démographiques des patientes et les détails du traitement étaient similaires dans la revue et dans notre série prospective : âge moyen (49 contre 54 ans, *p = *0,07), IMC (27 contre 27, *p = *0,47) et recours à la radiothérapie (76,5% vs 69%, *p = *0,79). La seule différence était la présence et l’implication du chirurgien plasticien pendant la phase extirpative pour identifier et préserver les vaisseaux. À notre institution, le chirurgien plasticien est présent tout au long de l’intervention et collabore étroitement avec le chirurgien oncologue dans une « danse » parfaitement coordonnée. Cette coordination n'a été décrite dans aucune des études examinées. **Conclusion :** Une implication coordonnée du chirurgien plasticien lors de la DGA pourrait diminuer le nombre de patientes « non reconstructibles » en RLI.

## Introduction

Breast cancer-related lymphedema (BCRL) following axillary lymph node dissection (ALND) remains a challenge for patients undergoing treatment for breast cancer, with BCRL occurring in as many as 60% of survivors.^1,2^ In order to try to prevent lymphedema, Bocadaro and colleagues first proposed the idea of immediate lymphatic reconstruction in 2009, whereby some lymphatic drainage is restored at the time of ALND by mapping, preserving, and anastomosing transected lymphatics in the axilla to nearby recipient veins.^3^ Since that time, this procedure has gained traction for breast and cutaneous malignancies with promising early results, including many retrospective comparative studies, prospective trials, and early randomized clinical trials.^1^^3–14^

While keeping abreast of advancements in this field, we were surprised by reports of patients planned for ILR, who did not undergo ILR due to a lack of available vessels in the axilla following ALND. Further reading into the methods of other studies, we noted that plastic surgical involvement in the ALND is highly variable, from joining the procedure immediately following the ALND to being present throughout, which we believe may contribute to the differences in vessel identification.^3,4,^^6–9^^,11,13,15,16^

At our institution, the plastic surgeon is a critical part of the entire ALND procedure in order to preserve lymphatics and veins, while obtaining complete oncologic clearance of nodes, in a well-coordinated “dance” with our oncologic surgery colleagues. This dance was rehearsed in the simulation lab and perfected in over a hundred cases of lymphatic reconstruction in many malignancies, in both groin and axillary node dissections, and the technique has been published.^17^ This study, therefore, aimed to investigate the impact of plastic surgery involvement throughout ALND and whether it may provide the opportunity for most patients to successfully undergo ILR.

## Methods

### Literature Review

In 2022, we published a systematic review of ILR in women with breast cancer undergoing ALND, which showed reduction of BCRL with ILR with a risk ratio of 0.22.^1^ To capture an updated number of patients planned for ILR, we utilized the same search parameters as the systematic review, expanding the date range up to 2024, while following PRISMA guidelines.^18^ Medline and Embase databases were searched for English language publications involving ILR from their inception up to the date noted above. The search strategy is shown in Supplemental Content 1. Papers included in our analysis were those that clearly stated the number of non-reconstructable patients who could not have ILR completed due to either a lack of available lymphatics, veins, or both. Abstracts, review articles and grey literature were excluded. From the included papers, ILR details were then extracted, which included the number of patients planned for ILR, the number of patients who successfully underwent ILR, and the reasons for patients not being able to have ILR completed. Additionally, we also extracted cancer diagnosis details, ALND details and treatment details when they were reported, as well as the extent of plastic surgical involvement in the identification and preservation of lymphatics and veins in the axilla. Where this data was ambiguous, we attempted to contact the corresponding authors for clarification.

### Chart Review

A chart review of prospectively maintained databases of the three senior plastic surgery authors (JM, AH, CTO) identified consecutive women undergoing ILR during ALND for breast cancer in accordance with our institution's ethics review board under the ethics ID HREBA.CC-22-0176. Extracted data included patient demographics, cancer diagnosis, ALND details including the number of nodes removed and the number of positive nodes, along with treatment details and the number of non-reconstructable patients.

### Lymphatic Preservation Technique

We have previously described our lymphatic mapping technique.^5,17,19^ In summary, dual lymphatic mapping was carried out prior to the ALND using both indocyanine green (ICG) injected into the ipsilateral first webspace as well as Patent Blue Dye (Guerbet, Canada) injected into the ipsilateral inner upper arm. One milliliter of ICG is injected and gently milked proximally to move the dye up the arm. The SPY-PHI Portable Handheld Imager (SPY-PHI; Stryker, MI, USA) device is used to visualize the lymphatic channels through the skin as they approach and enter the axilla. The oncologic surgeon then proceeds with the ALND. The node dissection proceeds medial to lateral (i.e., towards the arm), resulting in the lymph node “packet” hinged laterally, with blue/hot lymphatic channels entering from the arm.

The oncologic surgeon and plastic surgeon trade out operating at multiple time points. Once the medial pectoral nerve, long thoracic nerve, thoracodorsal nerve and axillary vein are identified, an additional vein is dissected emanating from the axillary vein more superficially to the thoracodorsal vein. This vein is traced distally to gain as much length as possible, requiring dissection around nodes in the nodal packet. Once the vein is clipped distally, the nodal tissue from level 1 and sometimes level 2 is brought laterally, at which point the plastic surgeon carefully dissects the blue lymphatics from the arm into the hinged lateral packet of nodes, continuing the dissection until a node is encountered, and then the lymphatic is divided and tagged with a small hemoclip and a 5 mm piece of 3-0 vicryl suture. Several such lymphatics are generally found. The oncologic surgeon then resumes operating, finishing the ALND by removing the node “packet,” leaving behind the preserved and tagged lymphatics.

### Data Analysis

The Mann‒Whitney *U* test was employed to examine differences between reconstructable and non-reconstructable groups in terms of demographics. Chi-square test was used to compare our data with the data from the literature. All statistical analyses were performed using Lifelines and Scipy libraries in Python (version 3.7.9).

## Results

### Literature Review

**Figure 1.**
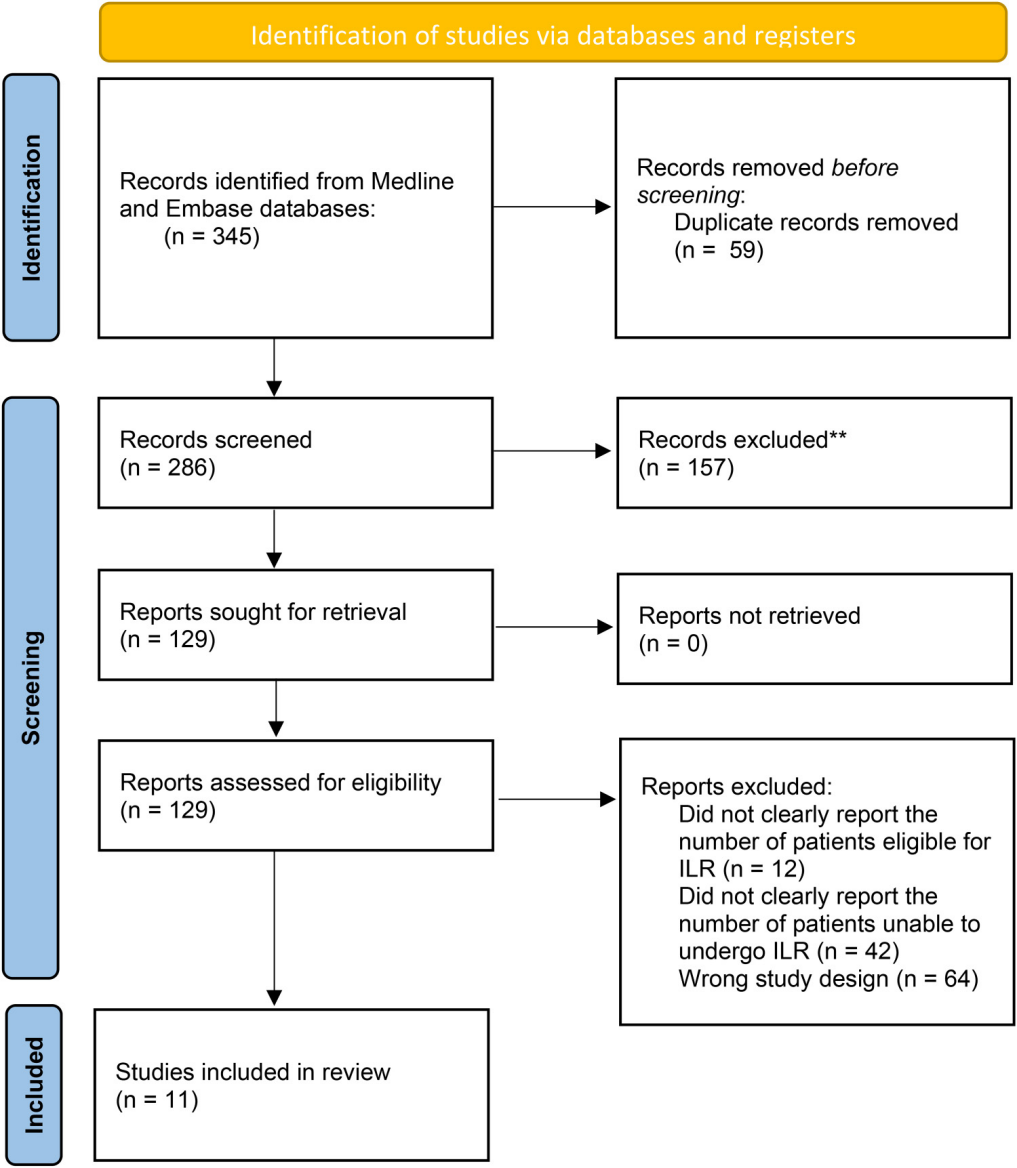
PRISMA flow diagram showing the results of the systematic review.

Our systematic review ultimately identified 11 publications which met inclusion criteria.^3,4,^^6–14^ The PRISMA flow diagram is shown in Figure 1. From these 11 studies, a total of 949 patients were consented to undergo ILR following ALND for breast cancer. Out of these patients, 133 or 14% could not have the procedure completed due to a lack of available veins or lymphatics. A summary of these findings is shown in Table 1. When available, demographic data was extracted and shown in Table 1. When it came to plastic surgical involvement in the procedure, there was variability among studies. While some reported a clear technique, others did not clearly state at which point plastic surgery became involved in the case, which is also detailed in Table 1.

**Table 1.** Results of the Systematic Review Which Identified 11 Papers That Clearly Reported the Number of Non-Reconstructable Patients.

Paper	Granoff et al. (2023)	Casabona et al. (2009)	Le et al. (2023)	Johnson et al. (2021)	Feldman et al. (2015)	Boccardo et al. (2009)	Levy et al. (2023)	Agrawal et al. (2018)	Boccardo et al. (2014)	Coriddi et al. (2024)	Schwarz et al. (2019)	Mean
Age	54	57	53	54	58.1	58	52.7	54.31	57	47.41	51.7	54.3
BMI	26.6	24.8	28.6	27.5	28.7	27.8	28.8	29.5	24	27.3	< 25 (25) 25–30 (18) 30.1–35 (7) > 35.1 (8)	27.4
Radiation	86.7	91.7	81.8	88	62.5	36.8	60	28.6			89	69.5
Number of patients consented for immediate lymphatic reconstruction	186	9	281	41	35	19	53	35	78	152	60	
Number of patients who were consented but could not have the procedure completed	28	8	29	9	8	1	8	0	3	44	2	
Procedure Details	Node dissection completed by surgical oncologist, lymphatic surgeon then inspected the axilla afterwards to identify vessels	Entire procedure completed by the plastic surgeon	Node dissections completed in conjunction with the oncologic surgeon	Node dissection completed by surgical oncologist, lymphatic surgeon then inspected the axilla afterwards to identify vessels	Not clear if done in conjunction, but methods state a clear attempt at preservation by clipping vessels close to the node packet	Unclear who performed the ALND, likely the plastic surgeon	Not clear if done in conjunction, but methods state a clear attempt at preservation by clipping vessels close to the node packet	Dye injected into the arm after the ALND which then completed by the oncologic surgeon. The plastic surgeon then injected dye and identified lymphatics	Unclear who performed the ALND, likely the plastic surgeon	Node Dissection completed by the oncologic surgeon	Entire procedure completed by the plastic surgeon	

### Our Data

Our retrospective chart review identified 68 patients who were planned for ILR. Of these 68 patients, 4 (5.9%) could not have the procedure completed due to a lack of available vessels. In three of these four cases, a lack of lymphatics for anastomosis was the reason ILR could not be performed. Prior to the ALND and after the injection of ICG, three of these four patients did not have clear lymphatics visible in the axilla. During the ALND, lymphatics could also not be visualized by either ICG nor with blue dye. A recipient vein was available in all four cases. The fourth patient unfortunately had a suspected reaction to either the blue dye or ICG, which led to respiratory concerns that required the cases to be completed quickly and for ILR to be abandoned. Patient demographics, cancer diagnosis, clinical staging and lymph node status are shown in Table 2. There were no significant differences in both patient demographics and cancer details between reconstructable and non-reconstructable patients.

**Table 2.** Results of the Chart Review Which Identified a Total of 68 Patients Who Underwent Attempted ILR.

		Overall	Reconstructable	Non-reconstructable	*P*-value	Test
*n*		68	64	4		
Age, median [Q1,Q3]		49.0 [45.0,58.5]	49.0 [45.0,58.5]	53.5 [48.2,59.2]	.624	Mann−Whitney
BMI, median [Q1,Q3]		26.7 [23.8,31.3]	26.7 [23.8,31.1]	29.3 [25.2,32.3]	.697	Mann−Whitney
Cancer type, *n* (%)	Ductal	60 (88.2)	57 (89.1)	3 (75.0)	.149	Fisher’s exact
	Inflammatory	1 (1.5)		1 (25.0)		
	Axillary adenocarcinoma of unknown primary	1 (1.5)	1 (1.6)			
	Lobular	6 (8.8)	6 (9.4)			
Stage, *n* (%)	Stage II	23 (33.8)	22 (34.4)	1 (25.0)	1	Fisher’s exact
	Stage III	36 (52.9)	33 (51.6)	3 (75.0)		
	Stage I	3 (4.4)	3 (4.7)			
	Stage IV	2 (2.9)	2 (3.1)			
	Unstagable	4 (5.9)	4 (6.2)			
Number of lymph nodes removed, median [Q1,Q3]		12.0 [10.0,18.2]	12.0 [9.8,18.2]	13.0 [11.5,18.8]	.589	Mann−Whitney
Number of positive lymph nodes, median [Q1,Q3]		2.0 [0.0,7.0]	2.0 [0.0,7.0]	2.0 [0.8,4.5]	1	Mann−Whitney
Chemotherapy, *n* (%)	Adjuvant	22 (34.9)	21 (35.0)	1 (33.3)	1	Fisher’s exact
	Neoadjuvant	22 (34.9)	21 (35.0)	1 (33.3)		
	Neoadjuvant and adjuvant	19 (30.2)	18 (30.0)	1 (33.3)		
Radiation, *n* (%)	Adjuvant	52 (76.5)	50 (78.1)	2 (50.0)	.233	Fisher's exact
	No radiation	16 (23.5)	14 (21.9)	2 (50.0)		
Hormone therapy, *n* (%)	No	19 (27.9)	16 (25.0)	3 (75.0)	.063	Fisher's exact
	Yes	49 (72.1)	48 (75.0)	1 (25.0)		

### Comparison

In comparison to the literature review, our collaborative approach to the axilla demonstrates a significantly lower percentage of non-reconstructable patients (5.9% vs. 14%; *p = .*03). In comparing patient demographic information, our patients had a similar average age (49y vs. 54y; *p = .*07) and the same average BMI (27 vs. 27; *p = .*47) to those in the literature. Similar to our patients, the majority of those in the literature received adjuvant radiotherapy (76.5% vs. 69%; *p = .*79).

## Discussion

ILR represents a positive step forward for breast cancer patients who require ALND. With growing evidence to support its efficacy, it is important to ensure that all patients eligible for the procedure can be reconstructed. In order to do so, preservation of lymphatics and veins in the axilla is essential. This concept has been briefly discussed in other papers, notably by Coriddi and colleagues, who identified that communication with the ablative surgeon is essential and scrubbing in for about five cases allows for the ablative surgeon to learn how to properly preserve veins.^16^ In our experience, scrubbing in for all cases provides even better preservation of vessels for anastomosis, particularly when it comes to preservation of adequate length of lymphatics, the dissection of which is much more familiar for microsurgeons. As noted in our data, a suitable vein for anastomosis was identified and preserved by the oncologic surgeon in all cases, without needing the plastic surgeon to be involved. Similar to Coriddi and colleagues, when we first began ILR, we assisted our oncologic surgery colleagues in their portion of the procedure and they quickly learned to preserve veins. We feel that the main reason behind our low numbers of non-reconstructable patients is therefore our collaborative preservation of lymphatics. This brief trade-off in operating surgeons allows us to dissect out lymphatics of adequate length while the node “packet” is still intact and hinged laterally on the arm end of the dissection, for a successful LVA with minimal disruption to the flow of the ALND. In a standard ALND, the lymphatics that it contains would be typically removed with the nodal specimen. In this situation, reconstructive surgeons who join at this portion of the case could then be faced with lymphatics too short to perform an LVA.

While this method of vessel preservation allowed for reconstruction in almost all cases, four of our patients still could not be reconstructed. On further analysis of these four patients, one of them actually had a suspected allergic reaction to either the ICG or Patent Blue Dye which caused some respiratory concerns raised by the attending anesthetist. Due to the need to quickly finish the case following the ALND, ILR was not attempted due to patient safety. Interestingly the remaining 3 “non-reconstructable” patients all had some degree of established pre-operative lymphedema noted by their reconstructive surgeon prior to their ALND and ILR, possibly secondary to scarring from their prior sentinel lymph node biopsy or due to axillary metastases. On further review of the literature, patients with pre-established lymphedema such as these are typically excluded from ILR trials and perhaps could have also been excluded from our study.^4,8,11^ Due to the lack of research on such patients, however, we opted to include them for completeness. Without these four patients with either an intraoperative allergic reaction or with pre-established lymphedema, our collaborative approach to the standard ILR patient would have resulted in a 0% “non-reconstructable” rate, further supporting its efficacy.

While an excellent method to ensure adequate vessels, there are some drawbacks to our collaborative approach to vessel preservation. First, the plastic surgeon must be present and scrubbed for most of the ALND, requiring a precise coordination of schedules and an investment of time by both surgeons. In certain centres with different pay structures, this method may not be financially viable for the reconstructive surgeon, who may otherwise be completing their own cases and joining the ILR case for the LVA portion of the procedure. Second, the operator swap and tagging of vessels adds more time to the ALND, thus increasing overall operative time and overall cost of the case. Despite this direct increase in time and cost, the addition of ILR for node positive breast cancer patients has already been found to be cost-effective overall by Johnson and colleagues, in their cost-utility analysis.^20^ In preventing lymphedema, patients no longer require lifelong visits to lymphedema clinics, therapy appointments, compression garments, time off of work to make appointments, use of emergency departments and office visits for cellulitis, making prevention ultimately money saving. Along with the cost benefits, we cannot underestimate the value of improved quality of life for patients for whom lymphedema is prevented.^21^ As the future demand for ILR increases, these barriers may however become a challenge in allowing as many patients as possible to access the procedure, given the need for such precise schedule coordination and time commitment. In the future, as our number of collaborative cases with our general surgery colleagues increases, it is certainly possible that they will gain the skills needed to preserve vessels without the reconstructive surgeon present, thus eliminating the need for both surgeons to be present for the ALND.

An additional criticism of plastic surgical involvement in the ALND is the thought of disrupting the oncologic completeness of node removal by dissecting out the lymphatics from the lymph node basin. It is, however, important to note that our dissection of the lymphatics only involves preserving length of the lymphatics up to the node, which we can see clearly with loupes. Additionally, we only preserve lymphatics from the arm, not those entering the axilla medially from the breast.

Limitations of this study include differential reporting of patient data and outcomes in the literature which led to overall less data comparison between our patients and review patients. Reporting of plastic surgeon involvement in ILR was highly variable between papers, making this portion of the analysis difficult. While some very clearly reported the role of the plastic surgeon, others did not specify this at all in their methods (Table 1). Interestingly, Agrawal and colleagues reported joining the case and identifying lymphatics after the completion of the ALND, but were able to identify vessels and perform ILR in all cases.^12^ Casabona reported performing the ALND themselves and similarly were able to perform ILR in all but one case.^7^ In Coriddi and colleagues’ clinical trial, which is the only RCT available to date, the plastic surgeon joined at the end of the ALND, which resulted in an almost 30% non-reconstructable rate.^4^ While some studies such as these clearly reported plastic surgical involvement, the variability of reporting in the other included studies certainly limited the strength of the statistical analysis.

## Conclusions

As the evidence for ILR grows, ensuring that as many eligible patients as possible are able to have ILR successfully performed is essential. Our collaborative approach to vessel identification and preservation has resulted in a very low number of patients who have failed ILR.

## Supplemental Material

sj-docx-1-psg-10.1177_22925503251404050 - Supplemental material for Women Planned for Immediate Lymphatic Reconstruction During Axillary Lymph Node Dissection Should Be Reconstructable: Improving Intraoperative Team Collaboration Supplemental material, sj-docx-1-psg-10.1177_22925503251404050 for Women Planned for Immediate Lymphatic Reconstruction During Axillary Lymph Node Dissection Should Be Reconstructable: Improving Intraoperative Team Collaboration by Spencer Yakaback, Rosalie Morrish, Golpira Elmi Assadzadeh, Antoine Bouchard-Fortier, Alexandra Hatchell, Jennifer Matthews and Claire Temple-Oberle in Plastic Surgery
