# Supplementary material for: Women Planned for Immediate Lymphatic Reconstruction During Axillary Lymph Node Dissection Should Be Reconstructable: Improving Intraoperative Team Collaboration
Source: Plast Surg (Oakv). 2025 Dec 19:22925503251404050. Online ahead of print. doi: 10.1177/22925503251404050 (PMC12716974; doi:10.1177/22925503251404050)
Supplement: sj-docx-1-psg-10.1177_22925503251404050 - Supplemental material for Women Planned for Immediate Lymphatic Reconstruction During Axillary Lymph Node Dissection Should Be Reconstructable: Improving Intraoperative Team Collaboration [file sj-docx-1-psg-10.1177_22925503251404050.docx]

SC 1 - Appendix: Search Strategy

1) anastomosis, surgical/ or microsurgery/

2) lymphatic system/ or lymph/ or lymphatic vessels/ or endothelium, lymphatic/ or lymphoid

tissue/ or lymph nodes/ or sentinel lymph node/

3) 1 and 2

4) ((lymphaticoven* or lymphovenous or lymphatic vessel*) adj2 anastomosis).tk,kw.

5) LVA.tw.kf.

6) 3 or 4 or 5

7) prophylactic surgical procedures/

8) (prophyla* or prevent* or immediate).tw,kw.

9) 7 or 8 ; 10) 6 and 9.
